# Development of Therapeutic Vaccines for Ovarian Cancer

**DOI:** 10.3390/vaccines8040657

**Published:** 2020-11-05

**Authors:** Stephanie Chow, Jonathan S. Berek, Oliver Dorigo

**Affiliations:** Department of Obstetrics and Gynecology, Division of Gynecologic Oncology, Stanford Women’s Cancer Center, Stanford Cancer Institute, Stanford University School of Medicine, Stanford, CA 94305, USA; stephchow@stanford.edu (S.C.); jberek@stanford.edu (J.S.B.)

**Keywords:** ovarian cancer, vaccines

## Abstract

Ovarian cancer remains the deadliest of all gynecologic malignancies. Our expanding knowledge of ovarian cancer immunology has allowed the development of therapies that generate systemic anti-tumor immune responses. Current immunotherapeutic strategies include immune checkpoint blockade, cellular therapies, and cancer vaccines. Vaccine-based therapies are designed to induce both adaptive and innate immune responses directed against ovarian cancer associated antigens. Tumor-specific effector cells, in particular cytotoxic T cells, are activated to recognize and eliminate ovarian cancer cells. Vaccines for ovarian cancer have been studied in various clinical trials over the last three decades. Despite evidence of vaccine-induced humoral and cellular immune responses, the majority of vaccines have not shown significant anti-tumor efficacy. Recently, improved vaccine development using dendritic cells or synthetic platforms for antigen presentation have shown promising clinical benefits in patients with ovarian cancer. In this review, we provide an overview of therapeutic vaccine development in ovarian cancer, discuss proposed mechanisms of action, and summarize the current clinical experience.

## 1. Introduction

Ovarian cancer is the deadliest of all gynecologic malignancies, with an estimated incidence of 11.4 per 100,000 women and death rate of 6.9 per 100,000 women [1]. Globally, approximately 295,000 women are diagnosed yearly with mortality reaching almost 185,000 [2]. Effective screening strategies to detect early stages of ovarian cancer are lacking, thus 75% of women are diagnosed at an advanced stage with a 46% survival five years after diagnosis [3].

Ovarian cancer treatment and management is typically comprised of surgery and chemotherapy. Primary treatment involves a hysterectomy with bilateral salpingo-oophorectomy, comprehensive surgical staging, and debulking followed by adjuvant platinum-based chemotherapy. For patients deemed poor surgical candidates or those with a low likelihood of optimal cytoreduction, neoadjuvant chemotherapy with potential interval debulking surgery is an option [3]. Over 80% of patients will respond to initial therapy, however the majority ultimately recur and require additional therapy. The development of chemotherapy-resistant disease over the course of often multiple lines of therapy is one of the major obstacles in the treatment of recurrent ovarian cancer. This highlights the need for new therapeutic interventions, including the development of immunotherapy for treatment of ovarian cancer [4].

Immunotherapy encompasses several interventions including cancer vaccines, immune checkpoint blockade, and adoptive cell therapy with the goal of enhancing tumor recognition by the immune system and immune effector-mediated tumor cell killing [5]. This multistep process involves the priming and activation of immune effector cells, in particular cytotoxic T cells. Tumor-infiltrating lymphocytes (TILs) can be found within the ovarian tumor microenvironment and are associated with improved prognosis in ovarian cancer patients [6,7,8]. The microenvironment of ovarian cancer is highly immune-suppressive and can effectively inhibit anti-tumor T cell responses. Various immune resistance mechanisms have been studied and include suppression of CD8+ and CD4+ effector cells by regulatory T cells (Tregs) [7,9], interruption of T cell proliferation by the immunoregulatory enzyme indoleamine-2,3-dioxygenase (IDO) [10,11], upregulation of inhibitory PD-L1 receptors [12,13], and production of myeloid derived suppressor cells [14] and cytokines (i.e., TGF-b) that impede antitumor immunity [15]. The extensive mechanisms by which ovarian tumors suppress antitumor immunity are important barriers to understand and overcome. Furthermore, recent data demonstrate significant intra-patient heterogeneity between different tumor sites in regards to patterns of T cell infiltration, T cell receptor repertoires, and immune infiltrates [16]. This heterogeneity presents an additional challenge to anti-tumor immune responses in patients with ovarian cancer as the disease is typically multifocal.

## 2. Cancer Vaccines

The concept of utilizing effector T cells to recognize antigen targets for cancer treatment has been studied for over a century. The first attempts to stimulate a cancer patient’s immune system were performed by Dr. William Coley in 1891. Inactivated *Streptococcus pyogenes* and *Serratia marcescens* were injected intratumorally after observing sarcoma regression in a patient with erysipelas [17]. In 1954, Black and colleagues found a correlation between the degree of lymphocytic infiltration and survival in patients with gastric carcinoma [18]. The link between immune cell infiltration and cancer survival provided evidence that cancer cells could be killed by immune cells. In 1957, Burnet suggested that differences in antigens between cancer and normal cells may be utilized to stimulate effective immunological responses [19]. Cancer vaccines have since emerged as an immunotherapy strategy that induces immune responses against tumor cells by presenting tumor specific antigens to the host.

Tumor associated antigens are recognized by the immune system and can generate T cell specific responses. Human tumor antigens are classified into one or more of the following categories: (i) differentiation antigens, (ii) mutational antigens, (iii) amplification antigens, (iv) splice variant antigens, (v) glycolipid antigens, (vi) viral antigens, and (vii) cancer testis antigens (CTAs) [20]. In addition to the antigen classifications, vaccines are also categorized into different types based on their mechanism of action: (i) dendritic cells, (ii) oncolytic viruses, (iii) modified cancer cells that secrete inflammatory cytokines, (iv) DNA encoding tumor associated antigens, and (v) intratumoral attenuated viral vaccines.

## 3. Vaccines in Ovarian Cancer

**Figure 1:**
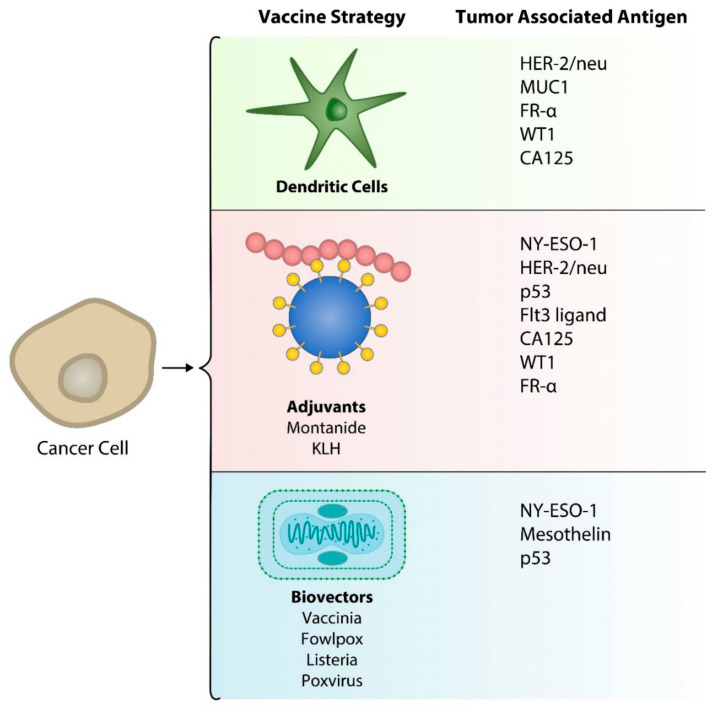
Strategies for presentation of tumor associated antigens in ovarian cancer vaccines.

Vaccines for ovarian cancer have been studied in various clinical trials over the last three decades, however the generation of vaccine-induced humoral and cellular immune responses have not shown significant anti-tumor efficacy. Recently, improvements in vaccine development have shown more promising clinical benefits in patients with ovarian cancer (Figure 1). Table 1 summarizes data from clinical trials that have reported on the clinical experience with vaccines in ovarian cancer patients. 

### 3.1. Dendritic Cell Vaccines

Dendritic cells (DCs) play a critical role in innate and adaptive immune responses. DCs are potent antigen presenting cells that capture and process antigens. Antigen presentation at local lymph node sites by dendritic cells stimulate antigen-specific cytotoxic T cells [63]. Vaccine development has sought to capitalize on the role DCs play in antitumor immunity. DCs pulsed with tumor-associated antigens have been shown effective as vaccine therapy in various cancer types [64].

Peptide-loaded and tumor lysate-loaded DCs are the two main strategies when using DCs as vaccines. Peptide-loaded DCs are pulsed with recombinant peptides prior to reinfusion. Data from various clinical trials have been published, providing positive efficacy signals (Table 1). Among these trials, Brossart and colleagues administered HER-2/neu or MUC1-derived peptide-pulsed dendritic cells in heavily pretreated metastatic breast and ovarian cancer patients [21]. One patient with ovarian cancer progression had a stable disease for over eight months while on therapy. Their study paved the way for additional peptide-pulsed DC vaccination therapies [22,24,27]. Loveland et al. used DCs pulsed with mannan-MUC1 fusion protein in 11 patients with adenocarcinomas. One ovarian cancer patient showed stable disease over three years of treatment [22]. Peethambaram et al. administered DCs loaded with recombinant HER-2/neu peptide and a granulocyte-macrophage colony-stimulating factor (GM-CSF) domain [24]. Two out of four ovarian cancer patients demonstrated stable disease over 15.7–18.3 months. WT1 peptide vaccines have had modest efficacy as demonstrated in various studies. The addition of low-dose cyclophosphamide prior to vaccination can potentially enhance vaccine potency [25]. In one study by Chu et al. using a HER-2/neu, hTERT, and PADRE peptide pulsed vaccine for maintenance therapy after treatment of recurrent ovarian cancer, 6 of 11 patients had no evidence of disease at 36 months, and the three-year progression-free survival was 80% with cyclophosphamide compared with 40% without. More recently, Gray et al. utilized a DC vaccine as maintenance therapy in epithelial ovarian cancer patients previously treated with one or two lines of conventional chemotherapy in complete remission [28]. CAN-003 was a phase 2b trial utilizing a MUC-1 protein-targeted DC vaccine. The treatment did not result in an increase in PFS or overall survival (OS), however patients in complete remission after second-line therapy were noted to have an improved OS with vaccination compared with controls (median OS 25.5 months with standard therapy vs. OS not yet reached with vaccination; HR 0.17; 95% CI 0.02–1.44; *p* = 0.07). DCs pulsed with neoantigen peptides have also been applied in the clinical setting [29,65]. Morisaki and colleagues administered a neoantigen peptide-pulsed DC in a case study of a woman with advanced stage ovarian cancer [29]. Following four rounds of vaccination, the patient had a significant decline in CA-125 levels with evidence of neoantigen-specific CTLs induced by vaccination.

DC vaccines electroporated with mRNA that subsequently is translated into protein have also been studied. Hernando and colleagues transfected DCs with mRNA-encoded folate-receptor-alpha (FR-α) [23]. Another study by Coosemans et al. loaded DCs with WT1 mRNA and found a two-month PFS and 64 month OS in their patient with serous epithelial ovarian cancer [26].

Whole tumor lysate-loaded DCs utilize whole tumor cells as a source of antigens, generating a variety of antigens associated with a specific tumor. In theory, using neoepitopes from tumor mutations will allow increased efficacy over single antigen vaccines. Bapsy et al. administered a whole tumor lysate-pulsed DC vaccine to 51 patients with advanced solid malignancies [33]. Of the seven ovarian cancer patients, one had a partial response and two had stable disease while on therapy. Hernando et al. vaccinated patients with advanced gynecologic malignancies with DCs pulsed with keyhole limpet hemocyanin (KLH) and autologous tumor cell lysate [30]. Mean progression-free interval while under vaccination was 25.5 months for patients with progressive or recurrent ovarian cancer.

Other studies have utilized personalized vaccines using autologous tumor lysate-loaded DCs and tumor antigen matched tumor cell lysates [31,32]. Tanyi et al. tested a personalized vaccine generated by autologous DCs pulsed with oxidized autologous whole-tumor cell lysate. The vaccine was injected into accessible lymph nodes in recurrent ovarian cancer patients and either administered alone, in combination with bevacizumab, or with bevacizumab plus low-dose intravenous cyclophosphamide. The treatment induced T cell responses to autologous tumor antigens and amplified T cell responses against mutated neoepitopes previously unrecognized. Overall survival of patients who showed vaccine treatment responses was 100% at 2 years compared with 25% in non-responders. Rob and colleagues provided encouraging evidence of a personalized dendritic cell vaccine (DCVAC) as maintenance therapy after primary debulking surgery and chemotherapy [66]. Interim analysis of his phase 2 trial demonstrated a 5.7-month improvement in PFS in patients receiving DCVAC sequentially after chemotherapy. Ongoing studies are underway to compare autologous oxidized tumor lysate loaded DCs with a ten peptide neoantigen based DC vaccine [65].

### 3.2. CTA Vaccines

Cancer testis antigen (CTA) are a type of differentiation antigen that is highly expressed in adult male germ cells with low expression in normal tissues and variably expression in tumor cells [67]. Among the over 70 cancer testis gene families identified as potential vaccine targets [67], NY-ESO-1 has been studied most extensively. NY-ESO-1 is a highly immunogenic tumor antigen that is expressed in up to 40% of ovarian cancer patients [68]. NY-ESO-1 expression in ovarian cancer is associated with a more aggressive phenotype, correlating with shorter PFS (22.2 vs. 25.0 months, *p* = 0.009) and OS (42.9 vs. 50.0 months, *p* = 0.002) [69].

NY-ESO-1 vaccination has been shown to elicit CD4+ and CD8+ T cell responses while demonstrating durable clinical responses [37,70]. Odunsi and colleagues conducted a phase I study of 18 women with NY-ESO-1-expressing ovarian cancers [35]. Patients immunized with the NY-ESO-1 derived peptide ESO157–170 had detectable ESO157–170-reactive CD4+ and CD8+ T cell responses, which correlated with a PFS of 19.0 months. Diefenbach et al. vaccinated “high-risk” ovarian cancer patients (suboptimal tumor debulking, failure of CA-125 to normalize after 3 cycles of chemotherapy, or positive second-look surgery) with NY-ESO-1b peptide and Montanide ISA-51, a vaccine adjuvant [36]. Median PFS was found to be 13 months. Sabbatini and colleagues investigated the use of overlapping long peptides from NY-ESO-1 in combination with two different vaccine adjuvants in ovarian cancer patients in second or third remission [37]. Of the 28 patients enrolled, 6 had no evidence of disease (NED) with a PFS range of 17–46 months. NY-ESO-1 is regulated by DNA methylation, and preclinical studies have demonstrated enhanced NY-ESO-1 expression and NY-ESO-1-specific CTL-mediated responses in ovarian cancer cell lines when treated with decitabine, a DNA methyltransferase inhibitor [71]. This observation provided the rationale for a clinical trial by Odunsi et al. in ovarian cancer patients. NY-ESO-1 vaccine, decitabine, and GM-CSF were administered to determine if epigenetic modulatory drugs improved antitumor response [38]. Of the 10 patients evaluable for clinical response, one had a partial response/disease remission and five had stable disease.

### 3.3. Protein/Peptide-Based Vaccines

Protein or peptide-based vaccines utilize defined tumor-associated antigens in conjunction with adjuvants. Tumor associated antigens are processed and presented to immune effector cells, in particular T cells, by host dendritic cells. Vaccines targeting HER-2/neu, p53, WT1, CA125, Flt3 ligand, and others have been studied in human clinical trials involving ovarian cancer.

One of the first proteins examined for an ovarian cancer vaccine therapeutic was HER-2/neu. Overexpression of the oncogene HER-2/neu is found in 15–30% of human adenocarcinomas [72]. Studies in humans have demonstrated that HER-2/neu MHC class I epitopes can induce interferon-γ-producing CD8+ T cells [39]. HER-2/neu protein immunization promotes native HER-2/neu immunity as well as antibody epitope spreading [72,73,74]. To date, there are various ongoing clinical trials involving HER-2/neu vaccine in ovarian cancer.

The tumor-suppressor protein p53 is overexpressed in almost all high grade serous ovarian cancer [75,76]. Antibodies against mutated p53 have been identified in approximately 25% of ovarian cancer patients [77]. Though induction of p53-specific immunity has been achieved with well-tolerated vaccines, the clinical efficacy has been modest thus far [40,41,43]. The overall lack of clinical benefit with a p53-specific vaccine prompted strategies for combination therapy with immunomodulatory agents. Chemotherapy, specifically cyclophosphamide, has been shown to suppress Treg function [78,79]. Treg cells in ovarian cancer have been shown to be a negative prognostic factor associated with decreased survival [80]. Vermeij et al. combined their p53-SLP vaccine with cyclophosphamide and demonstrated a 20% stable disease rate [78].

The WT1 protein is expressed in various solid cancers and hematologic malignancies, and has been ranked first in pilot prioritization of 75 cancer antigens [81,82]. The Wnt/β-catenin pathway has been implicated in the alteration of the ovarian cancer tumor microenvironment through immune cell modulation by improving DC, T cell, and macrophage function [83,84]. In ovarian cancer, WT1 expression is related to tumor type, grade, and stage, with WT1 expression highly associated with poor overall survival [85]. Ohno and colleagues administered a modified WT1 peptide vaccine to gynecological cancer patients with three out of 12 demonstrating stable disease [48]. In a phase II trial by Miyatake et al., 40 patients with gynecologic malignancies were given a WT1 peptide vaccine with 40% showing stable disease [49].

The CA125 antigen is a mucin-type glycoprotein associated with the cell membrane that has been routinely utilized as a clinical biomarker for screening and response to treatment in ovarian cancer [86]. It is a repeating peptide epitope of MUC16, which promotes malignant cell growth and inhibits anti-tumor immune responses [87]. In a large study of 119 advanced or recurrent ovarian carcinoma patients, Reinartz et al. utilized an anti-idiotypic antibody vaccine (ACA125) which mimics the CA125 antigen [45]. Overall, 68.1% were found to have an immunological response to the vaccine, with median OS of 19.4 months (range, 0.5–56.1 months). The subset of patients with antibodies to ACA125 had significantly longer survival times compared with negative responders (median 23.4 vs. 4.9 months, respectively).

The Flt3 receptor, a member of the receptor tyrosine kinase family, has also been proposed and studied as a potential vaccine antigen. In murine models, the Flt3 ligand enhances antigen-presenting cell function and stimulates natural killer cell precursor growth [44]. In a pilot study by Freedman and colleagues, the Flt3 ligand was administered to patients with ovarian cancer and mesothelioma via intraperitoneal and subcutaneous routes. Unfortunately, no objective responses were found.

Kalli and colleagues vaccinated ovarian and breast cancer patients with peptides based on folate receptor alpha, a tumor antigen expressed in a variety of cancers such as ovarian, breast, and lung [52]. Following vaccination, IFN-γ-producing T cells were enhanced, however no antibody responses were noted. All patients were alive at last follow-up of at least two years with a median relapse-free survival of 528 days in ovarian cancer patients in first remission and median survival was not reached for those in second remission.

The presentation of multiple peptides in a vaccine might theoretically increase the likelihood of generating T cells responses against a heterogenous tumor cell population and hence induce better anti-tumor responses compared to mono-valent vaccines [47,50]. A polyvalent vaccine conjugated with KLH and administered with OPT-821, an immunological adjuvant derived from the soapbark tree, was used in patients with ovarian, tubal, or primary peritoneal carcinoma of any stage [53]. Positive IgM responses were found in less than 50% of patients with median OS of 47 months.

Efforts have been made to customize cancer vaccines based on pre-existing tumor-specific antigens. Tsuda and colleagues reported on two regimens involving peptide vaccination in recurrent gynecologic cancers [46]. In their first study, patients were administered predesignated peptide vaccines, while the second study vaccinated patients with peptides to which preexisting peptide-specific cytotoxic T lymphocyte precursors in peripheral blood were confirmed. No clinical responses were found with the first regimen, however, in the second approach, seven out of 10 patients had enhanced peptide-specific cytotoxic T lymphocytes to additional peptides. Kawano and colleagues used a personalized peptide vaccine where antigens were selected based on pre-existing host immunity [51]. IgG responses were found augmented in 96.7% of patients following the 12th vaccination, however 31 of 37 cases showed disease progression, suggesting delayed tumor progression.

### 3.4. Recombinant Viral Vaccines

Viral vectors have been engineered to express multiple cancer antigens [88,89]. Jager et al. utilized recombinant vaccinia-NY-ESO-1 (rV-NY-ESO-1) and recombinant fowlpox-NY-ESO-1 (rF-NY-ESO-1) vaccines in patients with NY-ESO-1-expressing tumors [54]. In this study, patients were treated with rV-NY-ESO-1, rF-NY-ESO-1, or rV-NY-ESO-1 followed by rF-NY-ESO-1. One advanced ovarian cancer patient included in the cohort treated with rV-NY-ESO-1 had a clinical response and remained disease-free for 8 months following treatment. Odunsi and colleagues used rV-NY-ESO-1 and rF-NY-ESO-1 in advanced epithelial ovarian cancer and melanoma [57]. Of the 22 ovarian cancer patients, 42% had antibody seroconversions with spontaneous CD4+ T cell responses detected in 68% of patients. Fourteen percent had preexisting CD8+ T cell responses, and this increased to 45% post-vaccination.

Gulley and colleagues conducted a pilot study with PANVAC, a recombinant poxviral vaccine containing carcinoembryonic antigen (CEA) and MUC-1 transgenes in combination with 3 costimulatory molecules (B7.1, intracellular adhesion molecule-1, and lymphocyte function-associated antigen 3—collectively known as TRICOM).

The antigens were expressed by a vaccinia virus (PANVAC-V) for primary vaccination and fowlpox (PANVAC-F) for multiple booster vaccinations. Of the ovarian cancer patients treated with PANVAC, median PFS was 18 months (range, 2–19) and median OS was 19 (range, 6–21). A follow-up study using PANVAC in a heavily pre-treated cohort of metastatic breast and ovarian cancer with disease progression was reported by Mohebtash and colleagues [56]. In 14 ovarian cancer patients, median PFS was two months (range, 1–6 months) and median OS was 15.0 months (range, 1.5–57+ months).

More recently, Hardwick and colleagues evaluated a Modified Vaccinian Ankara vaccine delivering wild-type p53 (p53MVA) in platinum-resistant ovarian cancer [58]. Patients received a combination of p53MVA and gemcitabine. There was one partial response and three with stable disease with a median PFS of three months (range, 0.95–9.2 months). Five of the 11 patients demonstrated increased p53-reactive CD4+ and CD8+ T cells. In a subset analysis, there was a significant difference in median PFS between responders and non-responders (7.0 vs. 2.3 months, respectively).

## 4. Conclusions and Future Perspectives

Vaccine therapy for ovarian cancer has been studied in various clinical trials, and the development of new platforms and combinations with chemotherapy and adjuvants show promising clinical benefit (Table 2). There are still several challenges in creating safe and effective therapeutic cancer vaccines. The immunosuppressive and heterogenous tumor microenvironment in ovarian cancer remains a challenge. More studies are needed to improve vaccine-host interactions and to understand the variable immune responses to vaccine therapy. Other limitations include the labor-intensive protocols required to generate vaccines including surgical resection of tumor and the generation of autologous DCs. In addition, further studies are needed to determine the optimal indication for vaccine therapy. Maintenance therapy using vaccines to stimulate long-lasting immune system-mediated disease control might improve prognosis particularly in patients that do not derive a significant benefit from PARP inhibition. Novel research utilizing clustered regularly interspaced short palindromic repeats (CRISPR)-caspase 9 (Cas9) gene editing is currently underway, and the advent of more precise gene function alteration for therapy is on the horizon [90]. It is conceivable that continuous optimization of tumor antigen identification and presentation will lead to more effective therapeutic vaccines.

## Figures and Tables

**Table 1:** Summary of published clinical trials on ovarian cancer vaccines with clinical outcome to date.

Vaccine	Description	Total Patients (OC Patients)	Clinical Outcome *	Reference
DCs (peptide-pulsed)				
HER-2/neu or MUC1-derived peptide	Phase 1/2 study in heavily pretreated metastatic breast and ovarian cancer	10 (3)	1 SD over 8 months 1 SD over 8 weeks	Brossart et al., 2000 [21]
Mannan-MUC1	Phase 1 study in MUC1+ adenocarcinoma	11 (1)	1 SD	Loveland et al., 2006 [22]
mRNA-encoded FR-α	Pilot study in a patient with recurrent ovarian cancer	1	1 PR	Hernando et al., 2007 [23]
Lapuleucel-T, pulsed with BA7072, a recombinant fusion protein of HER-2/neu sequences linked to GM-CSF	Phase 1 study in HER-2/neu expressing metastatic breast, ovarian, and colorectal cancer	18 (4)	2 SD over 15.7–18.3 months	Peethambaram et al., 2009 [24]
HER-2/neu, hTERT, and PADRE	Phase 1/2 study in advanced ovarian cancer after first recurrence, randomized to receive low-dose cyclophosphamide prior to vaccination	11	6 NED at 36 months 3-yr PFS 80% 3-yr OS 100%	Chu et al., 2012 [25]
WT1 mRNA-loaded DC	Phase 1 study in epithelial ovarian carcinoma (OC) and ovarian carcinosarcoma (OCS)	2	OS 19 (OCS) and 12 (OC) months after drug cessation	Coosemans et al., 2013 [26]
Combinations of WT1, MUC1, and CA125	Retrospective study including patients with recurrent ovarian cancer	56	1-yr OS 87% 2-yr OS 65% 2 PR, 14 SD DCR 29% ORR 3.6%	Kobayashi et al., 2014 [27]
CVac, MUC-1 targeted DC	Phase 2b study (CAN-003 trial) in epithelial ovarian cancer as maintenance therapy	56	PFS 13 months CVac vs. 9 mo standard of care (HR 0.72, *p* = 0.33) Median OS 25.5 months with standard therapy vs. not yet reached with CVac (HR 0.17; 95% CI 0.02–1.44; *p* = 0.07)	Gray et al., 2016 [28]
Neoantigen peptides	Pilot study in a patient with advance ovarian cancer	1	CA-125 decreased from 4470 to 1303 U/mL. Patient expired approx. 1 year from treatment start	Morisaki et al., 2020 [29]
DCs (whole tumor lysate-pulsed)				
Pulsed with KLH and autologous tumor cell lysate	Phase 1 study in advanced gynecologic malignancies	8 (6)	PFI 25.5 months	Hernando et al., 2002 [30]
Pulsed with autologous tumor cell lysate supernatant	Pilot study in advanced ovarian cancer where patients were treated with metronomic cyclophosphamide and bevacizumab followed by vaccination	6	2 PR 2 SD	Kandalaft et al., 2013 [31]
DC pulsed with autologous hypochlorous acid-oxidized ov ca lysate	Pilot study in advanced ovarian cancer	5	2 SD 2 PD 1 mixed response	Chiang et al., 2013 [32]
APCEDEN, whole-tumor lysate pulsed DCs	Phase 2 study in refractory solid malignancies	51 (7)	1 PR 2 SD	Bapsy et al., 2014 [33]
Pulsed with oxidized autologous whole-tumor cell lysate	Pilot study in recurrent ovarian cancer using autologous vaccine with bevacizumab and cyclophosphamide	25	2 PR 14 SD	Tanyi et al., 2018 [34]
CTA				
ESO_157–170_	Phase 1 study in NY-ESO-1-expressing ovarian cancers	18	PFS 19.0 months	Odunsi et al., 2007 [35]
NY-ESO-1b peptide and Montanide ISA-51	Phase 1 study in “high-risk” ovarian cancer	9	PFS 13.0 months	Diefenbach et al., 2008 [36]
Synthetic overlapping long peptide from NY-ESO-1, Montanide ISA-51, and Poly-ICLC	Phase 1 study in advanced ovarian cancer in 2nd or 3rd remission	28	6 NED PFS range of 17–46 months	Sabbatini et al., 2012 [37]
NY-ESO-1, decitabine, and GM-CSF	Phase 1 study in relapsed ovarian cancer receiving doxorubicin as salvage therapy	12	1 PR 5 SD	Odunsi et al., 2014 [38]
Protein/Peptide				
HER-2/neu and GM-CSF	Phase 1 study in stage III or IV breast or ovarian cancer	6 (2)	Responses short-lived	Knutson et al., 2002 [39]
p53-SLP	Phase 2 study in recurrent epithelial ovarian cancer	20	2 SD	Leffers et al., 2009 [40]
p53-SLP	Long term outcomes of 2009 phase 2 study	20	RR 60.0% Median DSS 44.0 months	Leffers et al., 2012 [41]
p53-SLP with cyclophosphamide	Phase 2 study in recurrent ovarian cancer	10	2 SD	Vermeij et al., 2011 [42]
Wildtype p53 vaccine with Montanide and GM-CSF; p53-pulsed DC	Phase 2 study in high recurrence risk ovarian cancer. Two p53 vaccine approaches tested	13	Median OS 40.8 and 29.6 months arm A and B, respectively Median PFS 4.2 and 8.7 months, respectively	Rahma et al., 2012 [43]
Flt3 ligand	Pilot study in peritoneal carcinomatosis or mesothelioma patients	15 (9)	No objective responses	Freedman et al., 2003 [44]
Anti-idiotypic antibody vaccine (ACA125)	Phase 1/2b study in advanced ovarian cancer	119	Median OS 19.4 months (range 0.5–56.1 months) Ab3-positive patients had significantly longer survival time (median 23.4 mo, *p* < 0.0001) compared with Ab3-negative (median 4.9 mo)	Reinartz et al., 2004 [45]
Regimen 1: predesignated SART2 or ART4-derived peptide Regimen 2: peptides to which preexisting CTL precursor	Two regimens with different peptide vaccine regimens in recurrent gynecologic cancers	Regimen 1: 4 (2) Regimen 2: 10 (3)	Regimen 1: 0 response Regimen 2: 1 SD	Tsuda et al., 2004 [46]
Multipeptide vaccine with Montanide ISA-51 and GM-CSF	Phase 1 study in HLA-A1+, HLA-A2+, or HLA-A3+ epithelial ovarian, fallopian tube, or primary peritoneal carcinoma	9	DFS 19 months in 1 patient	Chianese-Bullock et al., 2008 [47]
WT1 peptide + Montanide ISA51	Phase 1 study in gynecological cancer patients with WT1/HLA-A *2402 positive tumors	12 (6)	1 SD	Ohno et al., 2009 [48]
WT1 peptide vaccine	Phase 2 study in progressive gynecologic cancers	40 (24)	10 SD OS HR 1.17 (95% CI 0.44–3.14; *p* = 0.75)	Miyatake et al., 2013 [49]
Multipeptide vaccine with Montanide ISA-51 and CM-CSF	Phase 1 study in HLA-A2+, stage II to IV epithelial ovaria, tubal, or primary peritoneal carcinoma after 1st or 2nd cytoreductive surgery with a complete clinical response	15 (8)	Median survival not reached	Morse et al., 2011 [50]
Personalized peptide vaccine (based on HLA-A types and IgG responses to peptides in pre-vaccinated plasma) with Montanide ISA-51	Phase 2 study in recurrent or persistent ovarian, fallopian tube, or primary peritoneal carcinoma	42	MST in platinum-sensitive vs. platinum-resistant 39.3 vs. 16.2 months, respectively. MST with monotherapy vs. in combination with chemotherapy in platinum-sensitive (39.3 vs. 32.2 months, respectively) and platinum-resistant (16.8 vs. 16.1 months, respectively)	Kawano et al., 2014 [51]
Folate receptor alpha with cyclophosphamide priming	Phase 1 study in stage II-IV ovarian cancer and stage II-III breast cancer without evidence of disease	22 (14)	All patients alive at last follow-up of at least 2 years Median RFS 528 days in patients in first remission Median OS not reached for those in second remission	Kalli et al., 2018 [52]
Polyvalent vaccine-KLH conjugate (including Globo-H-KLH, GM2-KLH, Tn-MUC1-32mer-KLH, TF-KLH) with adjuvant OPT-821	GOG 255 – Randomized, double-blinded, phase 2 study in any stage ovarian, fallopian tube, or primary peritoneal carcinoma in 2nd or 3rd complete remission. Patients were randomized to polyvalent vaccine-KLH conjugate + OPT-821 or OPT-821 alone (reference arm)	171	KLH + OPT-821 was not superior to OPT-821 alone (HR 0.98; 2-sided 95% CI, 0.71–1.36) Median OS for KLH + OPT-821 and OPT-821 were 47 and 46 months, respectively.	O’Cearbhaill et al., 2019 [53]
Recombinant Viral				
Recombinant vaccinia- and fowlpox-NY-ESO-1	Pilot study in advanced NY-ESO-1-expressing cancers	35 (1)	DFI 8 months	Jager et al., 2006 [54]
PANVAC	Pilot study in CEA- or MUC-1-expressing metastatic cancers	25 (3)	PFS range 2–19 months OS range 6–21 months	Gulley et al., 2008 [55]
PANVAC	Pilot study in metastatic ovarian and breast cancer with progressive disease	26 (14)	Median PFS 2 months (range 1–6 months) Median OS 15.0 months (range 1.5–57+ months)	Mohebtash, et al., 2011 [56]
Recombinant vaccinia- and fowlpox-NY-ESO-1	Two parallel phase 2 studies in NY-ESO-1-expressing epithelial ovarian cancer and melanoma	47 (22)	Median PFS 21 months (95% CI, 16–29 months) Median OS 48 months (95% CI, not estimable).	Odunsi et al., 2012 [57]
Modified Vaccinian Ankara vaccine delivering wildtype human p53 in combination with gemcitabine	Phase 1 study in platinum-resistant recurrent ovarian, fallopian tube, and primary peritoneal carcinoma	11	1 PR 3 SD Median PFS 3 months (range 0.95–9.2 months)	Hardwick et al., 2018 [58]
Whole tumor cell				
FANG, an autologous tumor-based vaccine containing a plasmid encoding GM-CSF and a novel bifunctional short hairpin RNA targeting furin convertase	Phase 1 study in advanced cancers	27 (5)	3 SD	Senzer et al., 2012 [59]
Live-attenuated				
ANZ-100, a live-attenuated Listeria vaccine and CRS-207, the live-attenuated Listeria strain expressing human mesothelin	Dual phase 1 study in treatment-refractory mesothelin-expressing cancers (mesothelioma, lung, pancreatic, ovarian) with hepatic metastases	9 (2)	No clinical responses	Le et al., 2012 [60]
Carbohydrate-based				
Theratope ^®^	Phase II/III study in advanced breast and ovarian cancer	70 (17)	Phase II (40 patients total): 27 patients relapsed (5 ovarian, 22 breast); 23 patients died (5 ovarian, 18 breast) Phase III (30 patients total): 18 patients relapsed (9 ovarian, 9 breast); 10 patients died (5 ovarian, 5 breast)	Holmberg et al., 2003 [61]
Lewis^y^-KLH conjugate with QS-21 adjuvant	Phase I study in recurrent or persistent ovarian, fallopian tube, or primary peritoneal carcinoma following primary therapy and were in complete clinical remission following additional chemotherapy	25	Median PFS 6 months 5 patients remained in complete clinical remission at 18 months follow up	Sabbatini et al., 2000 [62]

CR: complete response; CTA: cancer testis antigen; DCs: dendritic cells; DFI: disease-free interval; DFS: disease-free survival; DSS: disease-specific survival; GM-CSF: granulocyte macrophage colony-stimulating factor; HLA: human leukocyte antigen; HR: hazard ratio; hTERT: human telomerase reverse transcriptase; KLH: keyhole limpet hemocyanin; MST: median survival time; NED: no evidence of disease; OC: ovarian cancer; OS: overall survival; PADRE: pan-DR epitope; PFS: progression-free survival; PR: partial response; RFS: relapse-free survival; RR: response rate; SD: stable disease; SLP: synthetic long peptide; WT1: Wilms Tumor 1. * Outcomes correspond with ovarian cancer patients only.

**Table 2:** Ongoing (actively recruiting) trials utilizing ovarian cancer vaccines.

Trial	Vaccine	Clinical Trial Phase	Reference (ClinicalTrials.gov Identifier)
Ovarian Cancer Treatment With a Liposome Formulated mRNA Vaccine in Combination With (Neo-)Adjuvant Chemotherapy (OLIVIA)	W_ova1 vaccine, which includes 3 OC TAA RNAs	Phase 1	NCT04163094
Ovarian Dendritic Cell Vaccine Trial	DC vaccine made with autologous tumor lysate or for patients who are HLA-A2 with peptides of MUC1 and WT1 therapy	Phase 2	NCT00703105
Intensive Locoregional Chemoimmunotherapy for Recurrent Ovarian Cancer Plus Intranodal DC Vaccines	DC vaccine	Phase 1/2	NCT02432378
Study of Oncoimmunome for the Treatment of Stage III/IV Ovarian Carcinoma	OncoImmunome includes a mixture of 7–10 peptides identified based upon tumor-specific mutant peptide sequences from each tumor transcriptome	Phase 1	NCT02933073
Open Label Immunotherapy Trial for Ovarian Cancer (V3-OVA)	Tableted vaccine (V3-OVA) containing ovarian cancer antigens	Phase 2	NCT03556566
Phase 2 Study of Pembrolizumab, DPX-Survivac Vaccine and Cyclophosphamide in Advanced Ovarian, Primary Peritoneal or Fallopian Tube Cancer	DPX-Survivac	Phase 2	NCT03029403
Vaccine Therapy in Treating Patients With Metastatic Solid Tumors	Combination of 2 chimeric (Trastuzumab-like and Pertuzumab-like) HER-2 vaccine	Phase 1	NCT01376505
T-Cell Infusion, Aldesleukin, and Utomilumab in Treating Patients With Recurrent Ovarian Cancer	Aldesleukin, a recombinant human IL-2	Phase 1	NCT03318900
Arginase-1 Peptide Vaccine in Patients With Metastatic Solid Tumors	ARG1–18,19,20, an ARG1 peptide vaccine	Phase 1	NCT03689192
Phase Ib/IIa Trial to Evaluate Oregovomab and Nivolumab in Epithelial Cancer of Ovarian, Tubal or Peritoneal Origin (ORION-01)	Oregovomab, a murine monoclonal antibody against CA125	Phase 1/2	NCT03100006
P53MVA and Pembrolizumab in Treating Patients With Recurrent Ovarian, Primary Peritoneal, or Fallopian Tube Cancer	Modified vaccinia virus ankara vaccine expressing p53	Phase 2	NCT03113487
Autologous and Allogeneic Whole Cell Cancer Vaccine for Metastatic Tumors	Autologous or allogeneic tumor cells	Phase 1/2	NCT00722228
Galinpepimut-S in Combination With Pembrolizumab in Patients With Selected Advanced Cancers	galinpepimut-S, a WT1-targeting multivalent heteroclitic peptide vaccine	Phase 1/2	NCT03761914
DEC-205/NY-ESO-1 Fusion Protein CDX-1401, Poly ICLC, and IDO1 Inhibitor INCB024360 in Treating Patients With Ovarian, Fallopian Tube, or Primary Peritoneal Cancer in Remission	DEC-205/NY-ESO-1 Fusion Protein CDX-1401	Phase 1/2	NCT02166905
A Study of DSP-7888 Dosing Emulsion in Combination With Immune Checkpoint Inhibitors in Adult Patients With Advanced Solid Tumors	DSP-7888, a WT1 protein-derived peptide vaccine	Phase 1/2	NCT03311334

Tables OC: ovarian cancer; RNA: ribonucleic acid; DC: dendritic cell; ARG1: arginase-1; WT1: Wilms Tumor-1.

## References

[1] Henley S.J., Ward E.M., Scott S., Ma J., Anderson R.N., Firth A.U., Thomas C.C., Islami F., Weir H.K., Lewis D.R. (2020). Annual report to the nation on the status of cancer, part I: National cancer statistics. Cancer.

[2] Ferlay J., Colombet M., Soerjomataram I., Mathers C., Parkin D.M., Pineros M., Znaor A., Bray F. (2019). Estimating the global cancer incidence and mortality in 2018: GLOBOCAN sources and methods. Int. J. Cancer.

[3] Lheureux S., Gourley C., Vergote I., Oza A.M. (2019). Epithelial ovarian cancer. Lancet.

[4] Schaar B., Krishnan V., Tallapragada S., Chanana A., Dorigo O. (2019). Cell-based immunotherapy in gynecologic malignancies. Curr. Opin. Obstet. Gynecol..

[5] Chen D.S., Mellman I. (2017). Elements of cancer immunity and the cancer-immune set point. Nature.

[6] Hwang W.T., Adams S.F., Tahirovic E., Hagemann I.S., Coukos G. (2012). Prognostic significance of tumor-infiltrating T cells in ovarian cancer: A meta-analysis. Gynecol. Oncol..

[7] Sato E., Olson S.H., Ahn J., Bundy B., Nishikawa H., Qian F., Jungbluth A.A., Frosina D., Gnjatic S., Ambrosone C. (2005). Intraepithelial CD8+ tumor-infiltrating lymphocytes and a high CD8+/regulatory T cell ratio are associated with favorable prognosis in ovarian cancer. Proc. Natl. Acad. Sci. USA.

[8] Zhang L., Conejo-Garcia J.R., Katsaros D., Gimotty P.A., Massobrio M., Regnani G., Makrigiannakis A., Gray H., Schlienger K., Liebman M.N. (2003). Intratumoral T cells, recurrence, and survival in epithelial ovarian cancer. N. Engl. J. Med..

[9] Nishikawa H., Jager E., Ritter G., Old L.J., Gnjatic S. (2005). CD4+ CD25+ regulatory T cells control the induction of antigen-specific CD4+ helper T cell responses in cancer patients. Blood.

[10] Qian F., Liao J., Villella J., Edwards R., Kalinski P., Lele S., Shrikant P., Odunsi K. (2012). Effects of 1-methyltryptophan stereoisomers on IDO2 enzyme activity and IDO2-mediated arrest of human T cell proliferation. Cancer Immunol. Immunother..

[11] Qian F., Villella J., Wallace P.K., Mhawech-Fauceglia P., Tario J.D., Andrews C., Matsuzaki J., Valmori D., Ayyoub M., Frederick P.J. (2009). Efficacy of levo-1-methyl tryptophan and dextro-1-methyl tryptophan in reversing indoleamine-2,3-dioxygenase-mediated arrest of T-cell proliferation in human epithelial ovarian cancer. Cancer Res..

[12] Huang R.Y., Francois A., McGray A.R., Miliotto A., Odunsi K. (2017). Compensatory upregulation of PD-1, LAG-3, and CTLA-4 limits the efficacy of single-agent checkpoint blockade in metastatic ovarian cancer. Oncoimmunology.

[13] Matsuzaki J., Gnjatic S., Mhawech-Fauceglia P., Beck A., Miller A., Tsuji T., Eppolito C., Qian F., Lele S., Shrikant P. (2010). Tumor-infiltrating NY-ESO-1-specific CD8+ T cells are negatively regulated by LAG-3 and PD-1 in human ovarian cancer. Proc. Natl. Acad. Sci. USA.

[14] Khan A.N., Kolomeyevskaya N., Singel K.L., Grimm M.J., Moysich K.B., Daudi S., Grzankowski K.S., Lele S., Ylagan L., Webster G.A. (2015). Targeting myeloid cells in the tumor microenvironment enhances vaccine efficacy in murine epithelial ovarian cancer. Oncotarget.

[15] Sunde J.S., Donninger H., Wu K., Johnson M.E., Pestell R.G., Rose G.S., Mok S.C., Brady J., Bonome T., Birrer M.J. (2006). Expression profiling identifies altered expression of genes that contribute to the inhibition of transforming growth factor-beta signaling in ovarian cancer. Cancer Res..

[16] Zhang A.W., McPherson A., Milne K., Kroeger D.R., Hamilton P.T., Miranda A., Funnell T., Little N., de Souza C.P.E., Laan S. (2018). Interfaces of Malignant and Immunologic Clonal Dynamics in Ovarian Cancer. Cell.

[17] McCarthy E.F. (2006). The toxins of William, B. Coley and the treatment of bone and soft-tissue sarcomas. Iowa Orthop. J..

[18] Black M.M., Opler S.R., Speer F.D. (1954). Microscopic structure of gastric carcinomas and their regional lymph nodes in relation to survival. Surg Gynecol. Obstet..

[19] Burnet M. (1957). Cancer: A biological approach. III. Viruses associated with neoplastic conditions. IV. Practical applications. Br. Med. J..

[20] Odunsi K. (2017). Immunotherapy in ovarian cancer. Ann. Oncol..

[21] Brossart P., Wirths S., Stuhler G., Reichardt V.L., Kanz L., Brugger W. (2000). Induction of cytotoxic T-lymphocyte responses in vivo after vaccinations with peptide-pulsed dendritic cells. Blood.

[22] Loveland B.E., Zhao A., White S., Gan H., Hamilton K., Xing P.X., Pietersz G.A., Apostolopoulos V., Vaughan H., Karanikas V. (2006). Mannan-MUC1-pulsed dendritic cell immunotherapy: A phase I trial in patients with adenocarcinoma. Clin. Cancer Res..

[23] Hernando J.J., Park T.W., Fischer H.P., Zivanovic O., Braun M., Polcher M., Grunn U., Leutner C., Potzsch B., Kuhn W. (2007). Vaccination with dendritic cells transfected with mRNA-encoded folate-receptor-alpha for relapsed metastatic ovarian cancer. Lancet Oncol..

[24] Peethambaram P.P., Melisko M.E., Rinn K.J., Alberts S.R., Provost N.M., Jones L.A., Sims R.B., Lin L.R., Frohlich M.W., Park J.W. (2009). A phase I trial of immunotherapy with lapuleucel-T (APC8024) in patients with refractory metastatic tumors that express HER-2/neu. Clin. Cancer Res..

[25] Chu C.S., Boyer J., Schullery D.S., Gimotty P.A., Gamerman V., Bender J., Levine B.L., Coukos G., Rubin S.C., Morgan M.A. (2012). Phase I/II randomized trial of dendritic cell vaccination with or without cyclophosphamide for consolidation therapy of advanced ovarian cancer in first or second remission. Cancer Immunol. Immunother..

[26] Coosemans A., Vanderstraeten A., Tuyaerts S., Verschuere T., Moerman P., Berneman Z., Vergote I., Amant F., Van Gool S.W. (2013). Immunological response after WT1 mRNA-loaded dendritic cell immunotherapy in ovarian carcinoma and carcinosarcoma. Anticancer Res..

[27] Kobayashi M., Chiba A., Izawa H., Yanagida E., Okamoto M., Shimodaira S., Yonemitsu Y., Shibamoto Y., Suzuki N., Nagaya M. (2014). The feasibility and clinical effects of dendritic cell-based immunotherapy targeting synthesized peptides for recurrent ovarian cancer. J. Ovarian Res..

[28] Gray H.J., Benigno B., Berek J., Chang J., Mason J., Mileshkin L., Mitchell P., Moradi M., Recio F.O., Michener C.M. (2016). Progression-free and overall survival in ovarian cancer patients treated with CVac, a mucin 1 dendritic cell therapy in a randomized phase 2 trial. J. Immunother. Cancer.

[29] Morisaki T., Hikichi T., Onishi H., Morisaki T., Kubo M., Hirano T., Yoshimura S., Kiyotani K., Nakamura Y. (2020). Intranodal Administration of Neoantigen Peptide-loaded Dendritic Cell Vaccine Elicits Epitope-specific T Cell Responses and Clinical Effects in a Patient with Chemorefractory Ovarian Cancer with Malignant Ascites. Immunol. Invest..

[30] Hernando J.J., Park T.W., Kubler K., Offergeld R., Schlebusch H., Bauknecht T. (2002). Vaccination with autologous tumour antigen-pulsed dendritic cells in advanced gynaecological malignancies: Clinical and immunological evaluation of a phase I trial. Cancer Immunol. Immunother..

[31] Kandalaft L.E., Powell D.J., Chiang C.L., Tanyi J., Kim S., Bosch M., Montone K., Mick R., Levine B.L., Torigian D.A. (2013). Autologous lysate-pulsed dendritic cell vaccination followed by adoptive transfer of vaccine-primed ex vivo co-stimulated T cells in recurrent ovarian cancer. Oncoimmunology.

[32] Chiang C.L., Kandalaft L.E., Tanyi J., Hagemann A.R., Motz G.T., Svoronos N., Montone K., Mantia-Smaldone G.M., Smith L., Nisenbaum H.L. (2013). A dendritic cell vaccine pulsed with autologous hypochlorous acid-oxidized ovarian cancer lysate primes effective broad antitumor immunity: From bench to bedside. Clin. Cancer Res..

[33] Bapsy P.P., Sharan B., Kumar C., Das R.P., Rangarajan B., Jain M., Suresh Attili V.S., Subramanian S., Aggarwal S., Srivastava M. (2014). Open-label, multi-center, non-randomized, single-arm study to evaluate the safety and efficacy of dendritic cell immunotherapy in patients with refractory solid malignancies, on supportive care. Cytotherapy.

[34] Tanyi J.L., Bobisse S., Ophir E., Tuyaerts S., Roberti A., Genolet R., Baumgartner P., Stevenson B.J., Iseli C., Dangaj D. (2018). Personalized cancer vaccine effectively mobilizes antitumor T cell immunity in ovarian cancer. Sci. Transl. Med..

[35] Odunsi K., Qian F., Matsuzaki J., Mhawech-Fauceglia P., Andrews C., Hoffman E.W., Pan L., Ritter G., Villella J., Thomas B. (2007). Vaccination with an NY-ESO-1 peptide of HLA class I/II specificities induces integrated humoral and T cell responses in ovarian cancer. Proc. Natl. Acad. Sci. USA.

[36] Diefenbach C.S., Gnjatic S., Sabbatini P., Aghajanian C., Hensley M.L., Spriggs D.R., Iasonos A., Lee H., Dupont B., Pezzulli S. (2008). Safety and immunogenicity study of NY-ESO-1b peptide and montanide ISA-51 vaccination of patients with epithelial ovarian cancer in high-risk first remission. Clin. Cancer Res..

[37] Sabbatini P., Tsuji T., Ferran L., Ritter E., Sedrak C., Tuballes K., Jungbluth A.A., Ritter G., Aghajanian C., Bell-McGuinn K. (2012). Phase I trial of overlapping long peptides from a tumor self-antigen and poly-ICLC shows rapid induction of integrated immune response in ovarian cancer patients. Clin. Cancer Res..

[38] Odunsi K., Matsuzaki J., James S.R., Mhawech-Fauceglia P., Tsuji T., Miller A., Zhang W., Akers S.N., Griffiths E.A., Miliotto A. (2014). Epigenetic potentiation of NY-ESO-1 vaccine therapy in human ovarian cancer. Cancer Immunol. Res..

[39] Knutson K.L., Schiffman K., Cheever M.A., Disis M.L. (2002). Immunization of cancer patients with a HER-2/neu, HLA-A2 peptide, p369-377, results in short-lived peptide-specific immunity. Clin. Cancer Res..

[40] Leffers N., Lambeck A.J., Gooden M.J., Hoogeboom B.N., Wolf R., Hamming I.E., Hepkema B.G., Willemse P.H., Molmans B.H., Hollema H. (2009). Immunization with a P53 synthetic long peptide vaccine induces P53-specific immune responses in ovarian cancer patients, a phase II trial. Int. J. Cancer.

[41] Leffers N., Vermeij R., Hoogeboom B.N., Schulze U.R., Wolf R., Hamming I.E., van der Zee A.G., Melief K.J., van der Burg S.H., Daemen T. (2012). Long-term clinical and immunological effects of p53-SLP(R) vaccine in patients with ovarian cancer. Int. J. Cancer.

[42] Vermeij R., Leffers N., Hoogeboom B.N., Hamming I.L., Wolf R., Reyners A.K., Molmans B.H., Hollema H., Bart J., Drijfhout J.W. (2012). Potentiation of a p53-SLP vaccine by cyclophosphamide in ovarian cancer: A single-arm phase II study. Int. J. Cancer.

[43] Rahma O.E., Ashtar E., Czystowska M., Szajnik M.E., Wieckowski E., Bernstein S., Herrin V.E., Shams M.A., Steinberg S.M., Merino M. (2012). A gynecologic oncology group phase II trial of two p53 peptide vaccine approaches: Subcutaneous injection and intravenous pulsed dendritic cells in high recurrence risk ovarian cancer patients. Cancer Immunol. Immunother..

[44] Freedman R.S., Vadhan-Raj S., Butts C., Savary C., Melichar B., Verschraegen C., Kavanagh J.J., Hicks M.E., Levy L.B., Folloder J.K. (2003). Pilot study of Flt3 ligand comparing intraperitoneal with subcutaneous routes on hematologic and immunologic responses in patients with peritoneal carcinomatosis and mesotheliomas. Clin. Cancer Res..

[45] Reinartz S., Kohler S., Schlebusch H., Krista K., Giffels P., Renke K., Huober J., Mobus V., Kreienberg R., DuBois A. (2004). Vaccination of patients with advanced ovarian carcinoma with the anti-idiotype ACA125: Immunological response and survival (phase Ib/II). Clin. Cancer Res..

[46] Tsuda N., Mochizuki K., Harada M., Sukehiro A., Kawano K., Yamada A., Ushijima K., Sugiyama T., Nishida T., Yamana H. (2004). Vaccination with predesignated or evidence-based peptides for patients with recurrent gynecologic cancers. J. Immunother..

[47] Chianese-Bullock K.A., Irvin W.P., Petroni G.R., Murphy C., Smolkin M., Olson W.C., Coleman E., Boerner S.A., Nail C.J., Neese P.Y. (2008). A multipeptide vaccine is safe and elicits T-cell responses in participants with advanced stage ovarian cancer. J. Immunother..

[48] Ohno S., Kyo S., Myojo S., Dohi S., Ishizaki J., Miyamoto K., Morita S., Sakamoto J., Enomoto T., Kimura T. (2009). Wilms’ tumor 1 (WT1) peptide immunotherapy for gynecological malignancy. Anticancer Res..

[49] Miyatake T., Ueda Y., Morimoto A., Enomoto T., Nishida S., Shirakata T., Oka Y., Tsuboi A., Oji Y., Hosen N. (2013). WT1 peptide immunotherapy for gynecologic malignancies resistant to conventional therapies: A phase II trial. J. Cancer Res. Clin. Oncol..

[50] Morse M.A., Secord A.A., Blackwell K., Hobeika A.C., Sinnathamby G., Osada T., Hafner J., Philip M., Clay T.M., Lyerly H.K. (2011). MHC class I-presented tumor antigens identified in ovarian cancer by immunoproteomic analysis are targets for T-cell responses against breast and ovarian cancer. Clin. Cancer Res..

[51] Kawano K., Tsuda N., Matsueda S., Sasada T., Watanabe N., Ushijima K., Yamaguchi T., Yokomine M., Itoh K., Yamada A. (2014). Feasibility study of personalized peptide vaccination for recurrent ovarian cancer patients. Immunopharmacol. Immunotoxicol..

[52] Kalli K.R., Block M.S., Kasi P.M., Erskine C.L., Hobday T.J., Dietz A., Padley D., Gustafson M.P., Shreeder B., Puglisi-Knutson D. (2018). Folate Receptor Alpha Peptide Vaccine Generates Immunity in Breast and Ovarian Cancer Patients. Clin. Cancer Res..

[53] O’Cearbhaill R.E., Deng W., Chen L.M., Lucci J.A., Behbakht K., Spirtos N.M., Muller C.Y., Benigno B.B., Powell M.A., Berry E. (2019). A phase II randomized, double-blind trial of a polyvalent Vaccine-KLH conjugate (NSC 748933 IND# 14384) + OPT-821 versus OPT-821 in patients with epithelial ovarian, fallopian tube, or peritoneal cancer who are in second or third complete remission: An NRG Oncology/GOG study. Gynecol. Oncol..

[54] Jager E., Karbach J., Gnjatic S., Neumann A., Bender A., Valmori D., Ayyoub M., Ritter E., Ritter G., Jager D. (2006). Recombinant vaccinia/fowlpox NY-ESO-1 vaccines induce both humoral and cellular NY-ESO-1-specific immune responses in cancer patients. Proc. Natl. Acad. Sci. USA.

[55] Gulley J.L., Arlen P.M., Tsang K.Y., Yokokawa J., Palena C., Poole D.J., Remondo C., Cereda V., Jones J.L., Pazdur M.P. (2008). Pilot study of vaccination with recombinant CEA-MUC-1-TRICOM poxviral-based vaccines in patients with metastatic carcinoma. Clin. Cancer Res..

[56] Mohebtash M., Tsang K.Y., Madan R.A., Huen N.Y., Poole D.J., Jochems C., Jones J., Ferrara T., Heery C.R., Arlen P.M. (2011). A pilot study of MUC-1/CEA/TRICOM poxviral-based vaccine in patients with metastatic breast and ovarian cancer. Clin. Cancer Res..

[57] Odunsi K., Matsuzaki J., Karbach J., Neumann A., Mhawech-Fauceglia P., Miller A., Beck A., Morrison C.D., Ritter G., Godoy H. (2012). Efficacy of vaccination with recombinant vaccinia and fowlpox vectors expressing NY-ESO-1 antigen in ovarian cancer and melanoma patients. Proc. Natl. Acad. Sci. USA.

[58] Hardwick N.R., Frankel P., Ruel C., Kilpatrick J., Tsai W., Kos F., Kaltcheva T., Leong L., Morgan R., Chung V. (2018). p53-Reactive T Cells Are Associated with Clinical Benefit in Patients with Platinum-Resistant Epithelial Ovarian Cancer After Treatment with a p53 Vaccine and Gemcitabine Chemotherapy. Clin. Cancer Res..

[59] Senzer N., Barve M., Kuhn J., Melnyk A., Beitsch P., Lazar M., Lifshitz S., Magee M., Oh J., Mill S.W. (2012). Phase I trial of "bi-shRNAi(furin)/GMCSF DNA/autologous tumor cell" vaccine (FANG) in advanced cancer. Mol. Ther..

[60] Le D.T., Brockstedt D.G., Nir-Paz R., Hampl J., Mathur S., Nemunaitis J., Sterman D.H., Hassan R., Lutz E., Moyer B. (2012). A live-attenuated Listeria vaccine (ANZ-100) and a live-attenuated Listeria vaccine expressing mesothelin (CRS-207) for advanced cancers: Phase I studies of safety and immune induction. Clin. Cancer Res..

[61] Holmberg L.A., Oparin D.V., Gooley T., Sandmaier B.M. (2003). The role of cancer vaccines following autologous stem cell rescue in breast and ovarian cancer patients: Experience with the STn-KLH vaccine (Theratope). Clin. Breast Cancer.

[62] Sabbatini P.J., Kudryashov V., Ragupathi G., Danishefsky S.J., Livingston P.O., Bornmann W., Spassova M., Zatorski A., Spriggs D., Aghajanian C. (2000). Immunization of ovarian cancer patients with a synthetic Lewis(y)-protein conjugate vaccine: A phase 1 trial. Int. J. Cancer.

[63] Bates E.E., Dieu M.C., Ravel O., Zurawski S.M., Patel S., Bridon J.M., Ait-Yahia S., Vega F., Banchereau J., Lebecque S. (1998). CD40L activation of dendritic cells down-regulates DORA, a novel member of the immunoglobulin superfamily. Mol. Immunol..

[64] Sabado R.L., Balan S., Bhardwaj N. (2017). Dendritic cell-based immunotherapy. Cell Res..

[65] Sarivalasis A., Boudousquie C., Balint K., Stevenson B.J., Gannon P.O., Iancu E.M., Rossier L., Martin Lluesma S., Mathevet P., Sempoux C. (2019). A Phase I/II trial comparing autologous dendritic cell vaccine pulsed either with personalized peptides (PEP-DC) or with tumor lysate (OC-DC) in patients with advanced high-grade ovarian serous carcinoma. J. Transl. Med..

[66] Rob L., Mallmann P., Knapp P., Melichar B., Klat J., Minar L., Novotny Z., Bartunkova R., Spisek J., Pecen L. (2018). Dendritic cell vaccine (DCVAC) with chemotherapy (ct) in patients (pts) with epithelial ovarian carcinoma (EOC) after primary debulking surgery (PDS): Interim analysis of a phase 2, open-label, randomized, multicenter trial. J. Clin. Oncol..

[67] Almeida L.G., Sakabe N.J., de Oliveira A.R., Silva M.C., Mundstein A.S., Cohen T., Chen Y.T., Chua R., Gurung S., Gnjatic S. (2009). CTdatabase: A knowledge-base of high-throughput and curated data on cancer-testis antigens. Nucleic Acids Res..

[68] Odunsi K., Jungbluth A.A., Stockert E., Qian F., Gnjatic S., Tammela J., Intengan M., Beck A., Keitz B., Santiago D. (2003). NY-ESO-1 and LAGE-1 cancer-testis antigens are potential targets for immunotherapy in epithelial ovarian cancer. Cancer Res..

[69] Szender J.B., Papanicolau-Sengos A., Eng K.H., Miliotto A.J., Lugade A.A., Gnjatic S., Matsuzaki J., Morrison C.D., Odunsi K. (2017). NY-ESO-1 expression predicts an aggressive phenotype of ovarian cancer. Gynecol. Oncol..

[70] Jager E., Stockert E., Zidianakis Z., Chen Y.T., Karbach J., Jager D., Arand M., Ritter G., Old L.J., Knuth A. (1999). Humoral immune responses of cancer patients against "Cancer-Testis" antigen NY-ESO-1: Correlation with clinical events. Int. J. Cancer.

[71] Woloszynska-Read A., Mhawech-Fauceglia P., Yu J., Odunsi K., Karpf A.R. (2008). Intertumor and intratumor NY-ESO-1 expression heterogeneity is associated with promoter-specific and global DNA methylation status in ovarian cancer. Clin. Cancer Res..

[72] Disis M.L., Grabstein K.H., Sleath P.R., Cheever M.A. (1999). Generation of immunity to the HER-2/neu oncogenic protein in patients with breast and ovarian cancer using a peptide-based vaccine. Clin. Cancer Res..

[73] Disis M.L., Goodell V., Schiffman K., Knutson K.L. (2004). Humoral epitope-spreading following immunization with a HER-2/neu peptide based vaccine in cancer patients. J. Clin. Immunol..

[74] Disis M.L., Rinn K., Knutson K.L., Davis D., Caron D., dela Rosa C., Schiffman K. (2002). Flt3 ligand as a vaccine adjuvant in association with HER-2/neu peptide-based vaccines in patients with HER-2/neu-overexpressing cancers. Blood.

[75] Bartel F., Jung J., Bohnke A., Gradhand E., Zeng K., Thomssen C., Hauptmann S. (2008). Both germ line and somatic genetics of the p53 pathway affect ovarian cancer incidence and survival. Clin. Cancer Res..

[76] Nijman H.W., Lambeck A., van der Burg S.H., van der Zee A.G., Daemen T. (2005). Immunologic aspect of ovarian cancer and p53 as tumor antigen. J. Transl. Med..

[77] Soussi T. (2000). p53 Antibodies in the sera of patients with various types of cancer: A review. Cancer Res..

[78] Ghiringhelli F., Menard C., Puig P.E., Ladoire S., Roux S., Martin F., Solary E., Le Cesne A., Zitvogel L., Chauffert B. (2007). Metronomic cyclophosphamide regimen selectively depletes CD4+CD25+ regulatory T cells and restores T and NK effector functions in end stage cancer patients. Cancer Immunol. Immunother..

[79] Lutsiak M.E., Semnani R.T., De Pascalis R., Kashmiri S.V., Schlom J., Sabzevari H. (2005). Inhibition of CD4(+)25+ T regulatory cell function implicated in enhanced immune response by low-dose cyclophosphamide. Blood.

[80] Curiel T.J., Coukos G., Zou L., Alvarez X., Cheng P., Mottram P., Evdemon-Hogan M., Conejo-Garcia J.R., Zhang L., Burow M. (2004). Specific recruitment of regulatory T cells in ovarian carcinoma fosters immune privilege and predicts reduced survival. Nat. Med..

[81] Oka Y., Tsuboi A., Oji Y., Kawase I., Sugiyama H. (2008). WT1 peptide vaccine for the treatment of cancer. Curr. Opin. Immunol..

[82] Cheever M.A., Allison J.P., Ferris A.S., Finn O.J., Hastings B.M., Hecht T.T., Mellman I., Prindiville S.A., Viner J.L., Weiner L.M. (2009). The prioritization of cancer antigens: A national cancer institute pilot project for the acceleration of translational research. Clin. Cancer Res..

[83] Goldsberry W.N., Meza-Perez S., Londono A.I., Katre A.A., Mott B.T., Roane B.M., Goel N., Wall J.A., Cooper S.J., Norian L.A. (2020). Inhibiting WNT Ligand Production for Improved Immune Recognition in the Ovarian Tumor Microenvironment. Cancers.

[84] Haseeb M., Pirzada R.H., Ain Q.U., Choi S. (2019). Wnt Signaling in the Regulation of Immune Cell and Cancer Therapeutics. Cells.

[85] Carter J.H., Deddens J.A., Mueller G., Lewis T.G., Dooley M.K., Robillard M.C., Frydl M., Duvall L., Pemberton J.O., Douglass L.E. (2018). Transcription factors WT1 and p53 combined: A prognostic biomarker in ovarian cancer. Br. J. Cancer.

[86] Dochez V., Caillon H., Vaucel E., Dimet J., Winer N., Ducarme G. (2019). Biomarkers and algorithms for diagnosis of ovarian cancer: CA125, HE4, RMI and ROMA, a review. J. Ovarian Res..

[87] Felder M., Kapur A., Gonzalez-Bosquet J., Horibata S., Heintz J., Albrecht R., Fass L., Kaur J., Hu K., Shojaei H. (2014). MUC16 (CA125): Tumor biomarker to cancer therapy, a work in progress. Mol. Cancer.

[88] Hodge J.W., McLaughlin J.P., Kantor J.A., Schlom J. (1997). Diversified prime and boost protocols using recombinant vaccinia virus and recombinant non-replicating avian pox virus to enhance T-cell immunity and antitumor responses. Vaccine.

[89] Marshall J.L., Gulley J.L., Arlen P.M., Beetham P.K., Tsang K.Y., Slack R., Hodge J.W., Doren S., Grosenbach D.W., Hwang J. (2005). Phase I study of sequential vaccinations with fowlpox-CEA(6D)-TRICOM alone and sequentially with vaccinia-CEA(6D)-TRICOM, with and without granulocyte-macrophage colony-stimulating factor, in patients with carcinoembryonic antigen-expressing carcinomas. J. Clin. Oncol..

[90] He Z.Y., Zhang Y.G., Yang Y.H., Ma C.C., Wang P., Du W., Li L., Xiang R., Song X.R., Zhao X. (2018). In Vivo Ovarian Cancer Gene Therapy Using CRISPR-Cas9. Hum. Gene Ther..

